# Understanding transport processes in lichen, *Azolla*–cyanobacteria, ectomycorrhiza, endomycorrhiza, and rhizobia–legume symbiotic interactions

**DOI:** 10.12688/f1000research.19740.1

**Published:** 2020-01-23

**Authors:** Rahul Roy, Anke Reinders, John M Ward, Tami R McDonald

**Affiliations:** 1 Department of Plant and Microbial Biology, University of Minnesota, Minnesota, USA; 2 College of Continuing and Professional Studies, University of Minnesota, Minnesota, USA; 3 Biology Department, St. Catherine University, Minnesota, USA

**Keywords:** transporters, endomycorrhiza, ectomycorrhiza, rhizobia-legume, Azolla-cyanobacteria, lichens

## Abstract

Intimate interactions between photosynthetic and non-photosynthetic organisms require the orchestrated transfer of ions and metabolites between species. We review recent progress in identifying and characterizing the transport proteins involved in five mutualistic symbiotic interactions: lichens, *Azolla*–cyanobacteria, ectomycorrhiza, endomycorrhiza, and rhizobia–legumes. This review focuses on transporters for nitrogen and carbon and other solutes exchanged in the interactions. Their predicted functions are evaluated on the basis of their transport mechanism and prevailing transmembrane gradients of H ^+^ and transported substrates. The symbiotic interactions are presented in the assumed order from oldest to most recently evolved.

## Introduction

The vast majority of plant species participate in symbiotic relationships with fungi and bacteria. We know that the formation of symbiotic relationships between species occurred before the first eukaryotic cells which themselves are the product of endosymbiosis that resulted in the formation of mitochondria and chloroplasts. Understanding transmembrane transport processes between species is the key to understanding nutritional symbiotic interactions. This is because direct transfer from cell to cell between species is rare, although there are examples such as secretion between Agrobacterium and plant cells. In nutritional symbioses, cells of both species are bounded by a cell membrane and transfer of ions and metabolites occurs through membrane-bound transport proteins. Identifying the transporters involved and characterizing their activity can reveal which ions and metabolites are transported between species.

There has always been great interest in understanding symbiotic systems such as mycorrhiza and lichens, but research at the molecular level has often been too difficult. However, this is an appropriate time to revisit such complex systems and identify transport proteins that function in symbiotic interactions. Technical advances, especially the availability of genome sequences, have had an exceptionally large impact on the ability to study symbiotic interactions. Now, transcriptomic and proteomic experiments involving multiple interacting species can be performed and transporters can be assigned to each interacting partner. Once transporters are identified, heterologous expression experiments can be used to study transport activity, fluorescent protein fusions can be used to localize membrane proteins, and mutational analysis can be used to test whether transporter function is important for the interaction.

In the symbiotic associations discussed in this review, the transport of fixed nitrogen (N) and fixed carbon (C) compounds takes center stage. The reason is that all organisms need large amounts of N and C; photosynthetic plants can fix C, but only bacteria (and Archaea) can fix N. Cyanobacteria can fix both N and C but no eukaryotic organism can do the same. In the symbioses discussed here, large amounts of fixed N and C are transported between species. We will discuss transporters for N and C that have been identified and their activity and point out possibilities for transporters that have not yet been identified. We will also discuss selected transporters for other nutrients, ions, metals, and metabolites that are essential to symbiotic interactions.

To identify novel transporters functioning in a symbiosis, it is important to understand that the prevailing negative membrane potential, the transmembrane pH gradient that is more acidic outside, and the gradient of substrate may narrow the types of transporters likely to serve a particular function. For example, owing to the negative membrane potential, anion exporters are likely to be uniporters, such as anion channels. Exporters for cations are likely to be H ^+^-coupled antiporters or pumps. Anion uptake transporters are likely to be H ^+^-coupled symporters, and cation uptake transporters are likely to be H ^+^-coupled symporters or channels. For uncharged molecules, efflux transporters could be uniporters, H ^+^-coupled antiporters, or pumps, whereas uptake transporters are likely to be H ^+^-coupled symporters. It is also important to understand the orientation of membranes at the symbiotic interface. For all of the interactions discussed here, in order for ions and metabolites to be transferred from one organism to another, a transmembrane export step followed by an uptake step is required.

## Lichens

**Figure 1.**
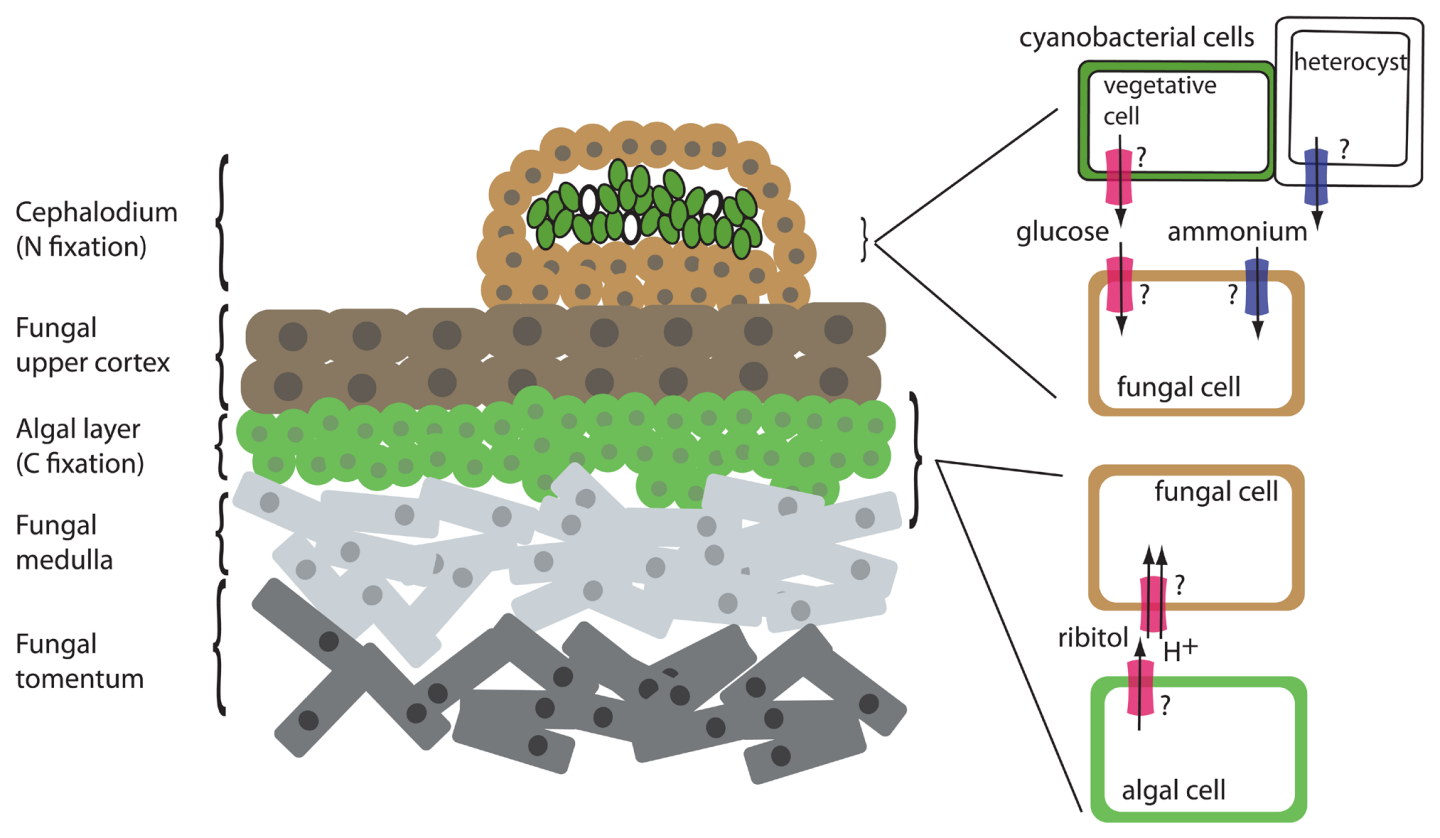
Transporters in the lichen symbiosis. Left: cross-section of a lichen thallus with both cyanobacterial and algal photobionts. Left: The cephalodium is a structure that houses the cyanobacterial cells. The cyanobacterial vegetative cells are shown in green, and the heterocysts are shown in white. “Tomentum” refers to the fungal area composed of closely matted mycelia. Right: Detail of symbiotic interface showing interaction of fungal cells with photosynthetic cells. Transporter activities that do not have an identified transporter are indicated with a question mark. Red transporter, C transporter (glucose or polyols such as ribitol); blue transporter, N transporter (ammonium).

Lichens are a morphologically and taxonomically diverse assemblage of obligate symbioses involving fungi and photosynthesizers. A typical lichen consists of a tough upper and lower fungal cortex protecting a symbiotic interaction zone consisting of a layer of algal or cyanobacterial cells supported by a network of fungal hyphae ( Figure 1). The entirety of this structure is called a thallus. Some lichens harbor two photosynthesizers, often a green alga as the main photobiont and a secondary cyanobacterial photobiont sequestered in wart-like growths called cephalodia. Close to 20% of currently described fungal species form lichens ^1, 2^.

Seminal radiolabeling experiments in the 1960s and 1970s established the nature of the carbohydrate transfer from the photosynthesizer to the fungus ( 3 and references therein). These experiments demonstrated that (1) a single C source is exported from the photobiont, (2) green algae transfer the polyol ribitol (in some cases, erythritol or sorbitol) whereas cyanobacteria transfer glucose, and (3) the transfer appears to be dependent on contact with, or signals from, the fungus, as the photobiont in pure culture does not excrete fixed C even as soon as 24 to 48 hours after isolation. Upon entry into the fungal cells, C is rapidly metabolized into mannitol, possibly through an intermediate such as arabitol. Cyanobacterial photobionts can also fix N into ammonia in specialized cells common to all multicellular cyanobacteria, called heterocysts or heterocytes, and transfer it to the fungus ^4^.

Genomes and transcriptomes are enabling a new understanding of the transfer of C and N in lichens. Genomes of several lichenizing fungi have been sequenced ^5– 11^. Although it is not yet possible to consistently reconstitute any mature lichen symbiosis in culture, the transcriptomes of the separated symbionts ^12, 13^, of the early stages of symbiotic interaction when cultures of the isolated symbionts are mixed ^14^, or of mature lichen symbioses in various metabolic states such as desiccation or rehydration ^15^ allow the identification of potentially important transporters of C and N. These excellent first forays set the stage for molecular dissection of the lichen symbiosis.

A current priority is testing of hypotheses flowing from genome and transcriptome experiments, such as functional characterization of the putative ribitol transporters identified by Yoshino *et al*. ^16^ in a lichenizing fungus. Interestingly, no exporter/importer pair for any of the symbiotically exchanged nutrients has yet been defined in any lichen system. Also missing from the discussion are sugar exporters in algae and cyanobacteria that could be *sugars will eventually be exported transporters* ( *SWEETs*) or *semiSWEETs*. Likewise, analysis of ammonium transporters (AMTs) and amino acid transporters as the conduits of symbiotically exchanged N is also necessary.

## *Azolla* 

The small aquatic fern *Azolla filiculoides* (and related species) has a mutualistic relationship with the cyanobacterium *Nostoc azollae* ( *Anabaena azollae*) and traditionally has been employed as a biofertilizer in rice paddies. More recently, there has been interest in using *Azolla* for bioremediation as a way to remove ammonium from wastewater or as a protein source ^17^. Azolla has also been implicated in the Arctic Azolla Event. Around 50 million years ago, exponential growth of the plant led to a rapid reduction of carbon dioxide (CO _2_) levels in the atmosphere, which is thought to have resulted in a cooling of the Earth ^18^. The genomes of both *A. filiculoides* and *N. azollae* have been sequenced ^19, 20^. The leaves of the fern contain cavities that are formed during development by invagination of the epidermis. Cyanobacteria are contained within these gas-filled cavities in close association with trichomes and embedded in a mucilaginous matrix. The structure of the trichomes is similar to that of transfer cells ^21^.

In contrast to the legume–rhizobia symbiosis, in *Azolla* the bacterial symbiont is transmitted from one generation to the next. Whereas the fern can grow without its symbiont, *N. azollae* is unable to exist outside of the plant. This is reflected in the genome of *N. azollae*, which has adapted to the symbiont lifestyle and, compared with related free-living cyanobacteria, appears reduced, and some core genes (for instance, that of phospho-fructokinase) have been lost ^19^.

Although *N. azollae* is able to perform photosynthesis, it does so only at a low rate and depends largely upon sugar derived from the plant ^22^. Much of the energy that *N. azollae* derives from the sugar it receives from *Azolla* is invested into N fixation. At the stage when N fixation is at its highest level, the bacteria are no longer dividing. The major product of photosynthesis in *Azolla* is sucrose ^22^, but it is not clear in what form C is exported from the plant and taken up into the cyanosymbiont.

**Figure 2.**
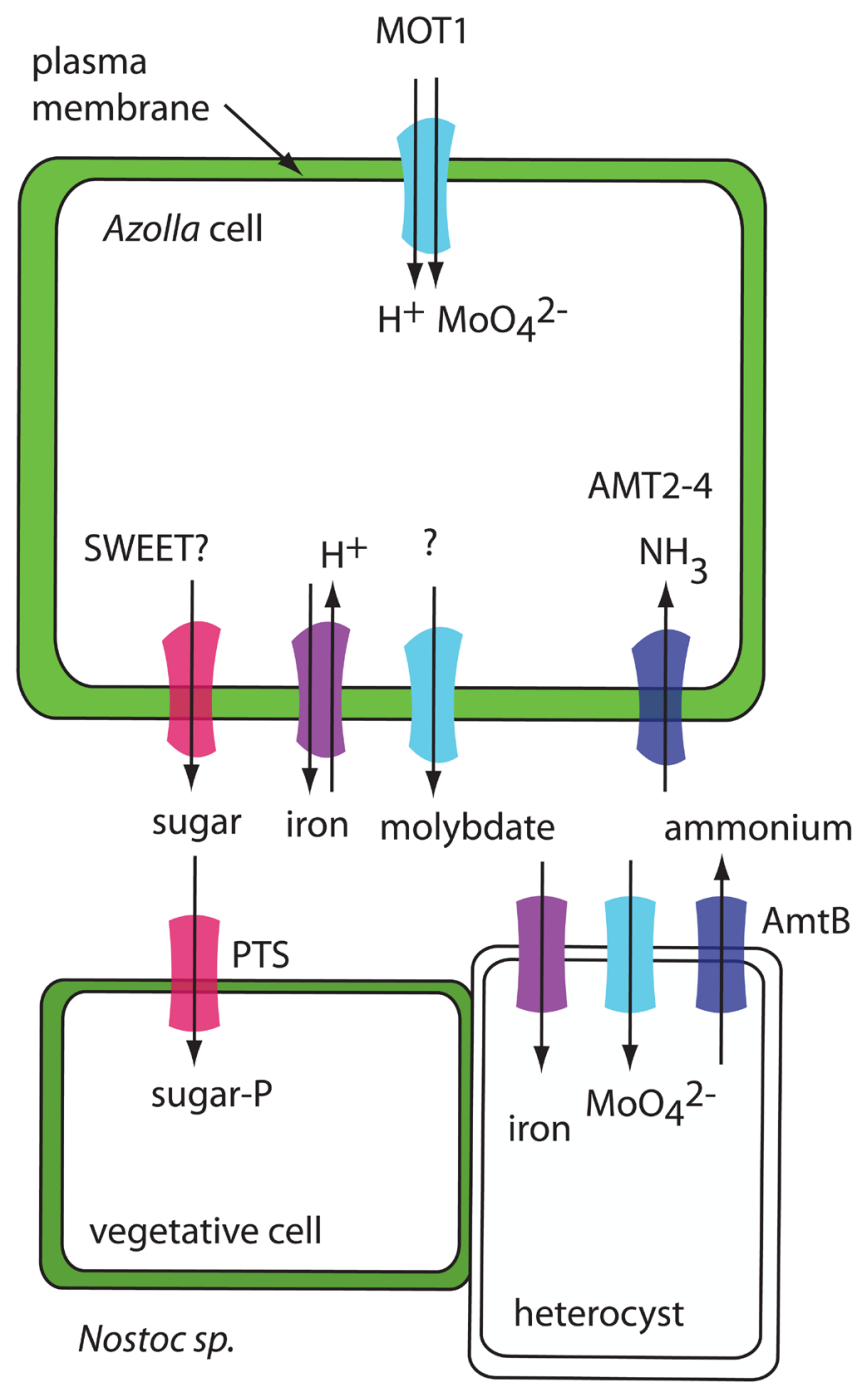
The Azolla–cyanobacteria symbiosis. In Azolla, expression of *MOT1*, *AMT2-4*, and an iron transporter related to *AtVIT1* is induced by N-limiting conditions. MOT1 may be an uptake transporter, AMT2-4 may transport fixed N from the cyanobacteria into the plant, and the iron transporter may serve as an iron efflux transporter. In the cyanobacteria, a phosphotransferase system (PTS) sugar transporter could function in sugar uptake and AmtB could function in ammonium efflux. Iron and molybdate transporters in the cyanobacteria are hypothetical.

In the bacteria, N fixation occurs in specialized cells, called heterocysts or heterocytes, which are more frequent in the symbiont than in related free-living *Nostoc sp.* ^23^. Fixed N is supplied to Azolla, and about 40% of the N is exported from the bacteria and released in the form of ammonium into the leaf cavity ^23^ ( Figure 2).

Even though it has been shown that Nostoc receives carbohydrates (at least some of it in the form of sucrose) from its plant partner ^22^, the necessary transporters of either symbiont have not been identified. The genome of *N. azollae* contains a gene for a phosphoenolpyruvate-dependent sugar phosphotransferase system (PTS). This type of transporter is common in bacteria and catalyzes the uptake and phosphorylation of a range of carbohydrates (for instance, glucose, fructose, or cellobiose) ^24^.

On the plant side, 15 *SWEET* genes are present in the *A. filiculoides* genome, but there is no evidence that any of them is differentially expressed in the presence of the cyanosymbiont ^25^. If SWEET proteins are involved in C effux in the *Azolla* symbiosis, the presence of the symbiont may trigger other post-transcriptional regulation.

*N. azollae* supplies its plant partner with ammonium, but no specific transporter responsible for this step has been identified. The genome of *N. azollae* contains an *AmtB* gene, encoding a putative ammonium transporter that could export ammonia into the leaf cavity of *Azolla*. Interestingly, *N. azollae* is lacking other N transporters (for instance, for nitrate or urea) ^19^. This may be an adaptation to the symbiont lifestyle, preventing *N. azollae* from obtaining N from the plant and making it dependent upon N fixation.

The genome of *A. filiculoides* contains genes for both electrogenic ( *AMT1*) and electroneutral ( *AMT2*) ammonium transporters ^17^. Expression of one of the *AMT2* genes, *AfAMT2-4* (Azfi_s0034.g025227.AMT2), is upregulated in the fern when no N is supplied in the growth medium but only in the presence of the cyanobiont ^25^. AMT2-4 is considered to function in the uptake of ammonium from the leaf cavity into the plant ( Figure 2).

The cyanobiont appears to be dependent on *Azolla* for additional nutrients. A molybdate transporter homolog, *AfMOT1* (Azfi_s0167.g054529), and an iron transporter homolog (Azfi_s0018.g014823) are induced in the fern under the same N-limiting conditions as *AMT2-4* ^25^. Both iron and molybdate are required co-factors for nitrogenase, the key enzyme for N fixation in the cyanobacteria. This suggests that, under N-limiting conditions, the plant upregulates Mo and Fe uptake transporters to supply more of these elements to the cyanobiont. AfMOT1 may function as a molybdate uptake transporter into the plant cells rather than as an exporter to the cyanobiont. The iron transporter Azfi_s0018.g014823 is related to vacuolar Fe/H ^+^ antiporter VIT1, so it could function in Fe export from plant cells if it is localized to the plasma membrane.

To fully understand the *Azolla/Nostoc* symbiotic interaction, the key transporters responsible for C efflux from the plant and N uptake into the plant need to be identified. With new technologies such as gene editing, it may be possible to test whether specific transporters such as AMT2-4 from *Azolla* are required for the interaction.

## Ectomycorrhiza

**Figure 3.**
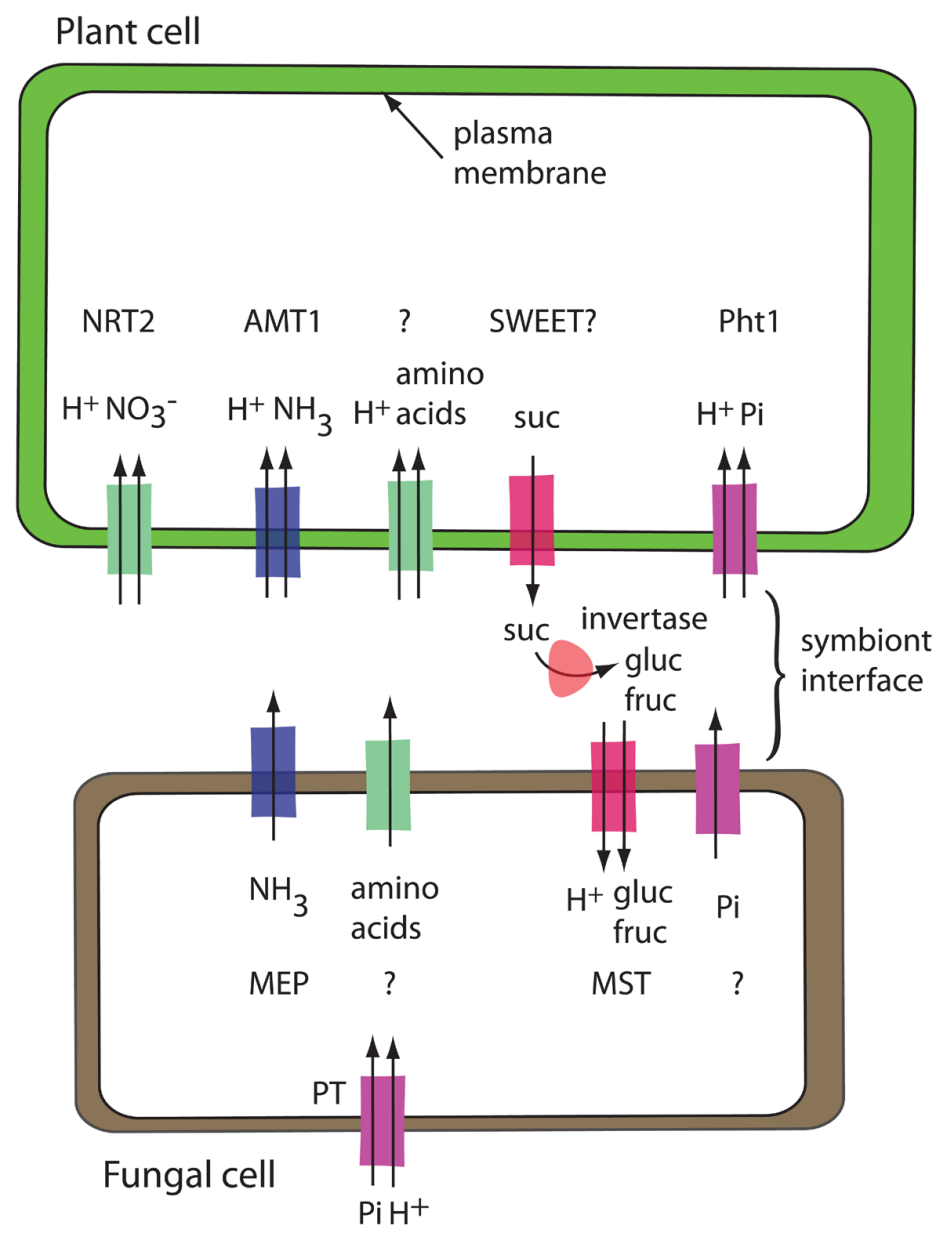
Model for ectomycorrhizal transporters. Transporters drawn with two arrows are H ^+^-coupled uptake symporters. Acid invertase, shown in the symbiont interface, is encoded by the plant. Transporters that are known are named, and transporters that have not been identified are indicated with a question mark. The fungal phosphate uptake transporter (PT) is shown away from the symbiont interface to emphasize that it is functioning in phosphate uptake from the environment rather than in phosphate export to the plant.

The ectomycorrhizal symbiosis consists of a plant partner, usually a tree species and a fungal partner, either an ascomycete or a basidiomycete. The fungus initially forms a dense layer of hyphae around the root tips of fine roots, called the mantle. Then hyphae penetrate between the cells of the epidermis and outer cortex of the tree root but without entering the cells. This structure is called the Hartig net. The fungal hyphae and the root cells share an apoplastic space, the symbiotic interface, a structure that is important for symbiosis ( Figure 3). The hyphae reach an extended area around the root of the tree, thereby allowing the tree to access nutrients and water beyond the reach of its roots ^26^.

The fungus receives fixed C from its plant partner in the form of hexoses. The tree exports sucrose into the wall space, where the sucrose is hydrolyzed by plant-derived acid invertase into glucose and fructose. Glucose is taken up preferentially ^27^. In the fungus, glucose is rapidly converted into trehalose and sugar alcohols such as mannitol or arabitol, allowing the fungus to continue to act as a sink for the long-distance transport of sugars ^27^.

In the ectomycorrhizal interaction, the plant benefits from metabolic capabilities of the fungus as well as the increased surface area for absorption from the soil. Interaction with the fungal partner enhances the supply of nutrients, in particular N and P ^28^. The form of N transported to the plant is not clear. Larsen *et al*. ^29^ concluded that amino acids are the main metabolites that the fungus synthesizes and shares with the plant. In their study, they found that expression of amino acid transporters is enriched in both plant and fungus, providing candidate genes to study in the interaction. Ammonium transporters have been identified in both plant and fungus. In poplar, a nitrate transporter in the NRT2 family is induced by the presence of the fungal partner ^30^. TbNrt2, a nitrate uptake transporter from the fungus *Tuber borchii*, is strongly expressed in the Hartig net and mantle and only weakly in free hyphae ^31^. There is no evidence for an enrichment of ammonium or inorganic phosphate (Pi) transporters in the ectomycorrhizal association ^29^. Three fungal H ^+^-coupled phosphate uptake transporters have been isolated from *Hebeloma*: HcPT1.1, HcPT1.2, and HcPT2 ^32– 34^. All are likely to function in Pi acquisition by the fungus. HcPT2 is localized at sites of both Pi uptake and Pi release, and RNA interference (RNAi) lines of *Hebeloma* with decreased expression of *HcPT2* form fewer ectomycorrhizae than the control strain ^33^. However, owing to its predicted transport mechanism and the existing proton gradient with higher proton concentration in the apoplast, it is unlikely that HcPT2 activity is responsible for Pi export to the plant. An additional transporter required for P efflux to the plant has not been identified yet.

It is generally accepted that the form of C taken up by the fungus is hexoses. Several hexose transporters, HXTs or MSTs in the major facilitator superfamily, have been isolated from a range of ectomycorrhizal fungi ^35^. They function as H ^+^-coupled uptake transporters ^36, 37^. None of them has been shown to be essential for symbiosis, but several are specifically expressed in the root tips or in Hartig net/mantle versus free-living hyphae. In an ectomycorrhizal transcriptome study, sugar transporters were found to be specifically enriched in the transcripts of the fungal partner. No such enrichment occurred in the plant transcriptome ^29^. This may indicate that the fungal partner drives the movement of sugars, at least as far as the transport step is concerned. The plant partner may control the supply of photosynthates through controlling the activity of acid invertase in the apoplast, thereby limiting the amount of hexoses available for the fungal partner to take up.

Although no specific transporters have been shown to be required for ectomycorrhizal symbioses, a lot of progress has been made in genome sequencing and transcriptomic experiments to identify a range of transporters that may be important. Export of sucrose is likely via SWEET proteins and uptake of hexoses by MSTs in the fungus. It would be very useful to know the forms of N and P that are taken up by the plant, and identifying transporters that are necessary for the interaction will help narrow the list of possibilities.

## Endomycorrhiza

**Figure 4.**
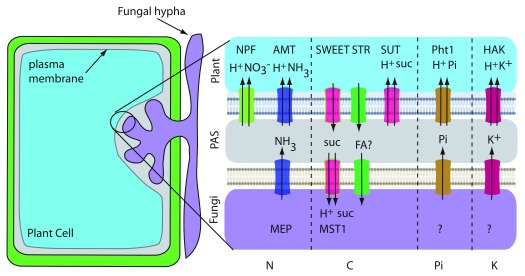
Transporters in endomycorrhizal or arbuscular mycorrhizal (AM) interactions. A plant cell containing an arbuscule is shown. Transporters that are known are named in the illustration. Transporters drawn with two arrows are H ^+^-coupled uptake symporters. Transporter activities that do not have an identified transporter are indicated with a question mark. PAS, periarbuscular space.

Endomycorrhizal or arbuscular mycorrhizal (AM) symbioses are ancient and widespread interactions between obligate biotrophic fungi belonging to the Glomeromycotina and the roots of land plants ^38^. This intimate association enables nutrient exchange, and plants provide up to 20% of the photosynthetically fixed C to the fungi and derive mineral nutrients from the fungal partner ^35, 39, 40^. Fungal hyphae grow between cells in the root cortex and through the cell wall surrounding plant cells. The fungi do not break through the plasma membrane; they form highly branched structures known as arbuscules in the apoplastic space ( Figure 4). The formation of arbuscules is a signal-based process involving significant transcriptional reprogramming and cellular remodeling ^41^. The arbuscule is surrounded by a plant-derived periarbuscular membrane (PAM), thus being physically separated from the host cytoplasm. Recent studies suggest that the PAM encloses a periarbuscular space (PAS) which contains many hyphal branches, challenging an earlier notion that the PAM trails along each hyphal branch ^42^. The arbuscule provides an increased surface area for exchange of nutrients between the two organisms. Membrane transport in AM symbiosis has been studied mainly on the plant side because of the difficulty of working with AM fungi and the complexity of fungal genomes. The AM fungi establish a new C sink in the roots of plants, thus driving the transport of photoassimilates or C reserves from the host plant, preferentially in the form of hexoses, primarily glucose. Studies reveal significant transcriptional upregulation of the members of MST, sucrose transporter (SUT) and SWEET families of transporters in the arbuscule-containing cells of many plant species, although most of the results await genetic and functional validation ^35, 40^. Recently, SWEET1b from *Medicago truncatula* was localized to the PAM and demonstrated to transport glucose ^43^. However, loss-of-function *mtsweet1b* mutants show no defect in the plant–fungal symbiotic interaction, indicating the presence of redundant transporters. Interestingly, *sut2* mutants in tomato reveal higher AM fungi colonization rates, suggesting a role for SUT2 in reverse transport of sucrose from the PAS space back to the plant ^44^ ( Figure 4). Sugar transporters in AM fungi are less well studied. GpMST1 from *Geosiphon pyriformis* and RiMST2 from *Rhizophagus irregularis* transport hexoses and have the highest affinity for glucose ^45, 46^. RiMST2 is expressed in arbuscules and intercellular hyphae where it may function in sugar uptake from the plant ^46^. Interestingly, active uptake of ^14^C-labelled sugars by the extraradical mycelium indicates that sugar transport by the fungus also occurs outside of arbuscules ^47^.

Lipids may serve as another source of C transferred from plants to AM fungi. AM fungi depend on the plant host for lipids since they lack genes encoding the cytosolic fatty acid synthase subunits for fatty acid biosynthesis ^48, 49^. This dependency was revealed by legume mutants defective in lipid biosynthetic genes that have stunted arbuscules and reduced colonization. Other studies using transgenic *Medicago* expressing fatty acid synthesis genes and radioisotopes also demonstrate the transfer of fatty acids from plants to fungi. The ABC-type transporters STR1 and STR2 are required for arbuscule formation in rice and *Medicago* ^50, 51^ and are predicted to transport beta-monoacylglycerol from plants to fungi ^52, 53^. The mechanism of transport or trafficking of lipids at the AM–plant interface is not clear and will require additional research such as using imaging with lipophilic dyes or transport assays using reconstitution of transporter proteins in liposomes.

Owing to the low mobility of phosphate ions in soil, plants use the extensive network of AM fungi to acquire most of their P. Availability of Pi is also a signal for arbuscule maintenance since plant Pi transporter mutants display premature degeneration of arbuscules ^54^ and exogenous Pi treatment in rice inhibits fungal colonization and mycorrhizal uptake of Pi ^55^. Krajinski *et al*. ^56^ revealed that Pi uptake by AM fungi is driven by the transmembrane H ^+^ gradient. Fungal Pi/H ^+^ symporters are responsible for Pi uptake from the soil by the extraradical mycelium, although they are also expressed in the intraradical mycelium, suggesting their role in reabsorption from the PAS. Pi is then trafficked as polyphosphate chains to the intraradical mycelium, where breakdown produces Pi which then gets transported into the PAS via a still-unknown mechanism. AM fungi colonization induces the expression of Pi transporters in rice ( *OsPT11*), tomato ( *LePT1*), potato ( *StPT3*), maize ( *ZmPT9*) and *Medicago truncatula* ( *MtPT4*) ^35, 40^. Both low- and high-affinity H ^+^/Pi symporters of the plant PHT1 family have been implicated in AM fungi symbioses and these cluster in a separate clade from PHT1 transporters in non-AM symbiotic plants. Plant and fungal Pi transporters might also function as transceptors sensing Pi, and further investigation is warranted ^57, 58^. Interestingly, high Pi represses *STR1/2* expression, suggesting that plants withhold C under high Pi conditions ^59^. For a detailed review of Pi transport in mycorrhiza, see ^60^.

N acquisition by AM fungi can provide plant partners with almost one third of their N needs ^61^. Fungi encode ammonium transporters in the MEP family that could serve as ammonium exporters ^62^, and high- and low-affinity MEPs have been reported in *R. irregularis.* AM-inducible *AMTs* are found in all host plants (both dicots and monocots) and have been localized to the PAM ^63^. Since the PAS is acidic, NH _4_^+^ concentration predominates over NH _3_. Plant AMTs are thought to bind NH _4_^+^, deprotonate it, and co-transport NH _3_ and H ^+^ ( Figure 4). Nitrate might also be transferred since plant nitrate transporters are induced by AM colonization ^35, 40^. Nitrate transporters are also induced by high phosphate or low nitrate concentrations, suggesting a complex regulation.

## Legume–rhizobia symbiosis

**Figure 5.**
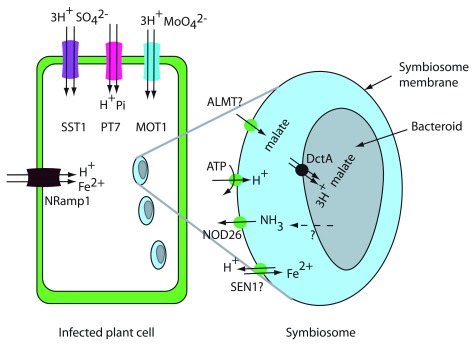
Legume–rhizobia transport processes. Plant transporters for malate efflux, iron efflux, and ammonium uptake are shown on the symbiosome membrane. At the plasma membrane of infected plant cells, SST1, MOT1, Nramp1, and PT7 are thought to function in uptake into the plant cells. In the bacteroid membrane, DctA, the bacterial C _4_-dicarboxylate transporter, is shown on the bacteroid membrane. NH _3_ diffusion through the bacteroid membrane is indicted by a dashed line.

In the rhizobia–legume interaction, the plant responds to signals produced by the bacterium and initiates a developmental program leading to the formation of a plant root structure called the nodule. Within the nodule, the modified rhizobia, called bacteroids, are housed in intracellular, membrane-bound compartments called symbiosomes. The plant symbiosome membrane (SM), also called the peribacteroid membrane (PBM), surrounds the bacteroids ( Figure 5). The plant supplies fixed C and a myriad of other ions, metabolites, and proteins to create a suitable environment for the bacteroids to fix N which is absorbed by the plant. Recent advances in proteomic and transcriptomic analysis have led to the identification of transporters in the PBM.

Transporters involved in the interaction of legumes and rhizobia have been recently reviewed in detail ^64, 65^. Despite their central importance for the symbiosis, most of the N and C transporters predicted to be required for the interaction have not been identified.

The PBM contains plasma membrane-type H ^+^ATPases that acidify the peribacteroid space and generate a negative membrane potential ^64^. To understand transport processes at the PBM, the peribacteroid space should be considered equivalent to the apoplast or cell wall space of a plant cell. Accordingly, solutes are transported from the plant to bacteroids via plant exporter proteins.

It is clear that the form of fixed C taken up by bacteroids is C _4_-dicarboxylates ^66^. Bacterial mutants defective in dicarboxylate transporter DctA are unable to fix N. Malate, succinate, and fumarate are transported by DctA ^66, 67^. Malate appears to be the most important and only required C _4_-dicarboxylate supplied to the bacteroids by the plant ^68^. It is not surprising that the uptake of dicarboxylates by DctA is coupled to the uptake of at least three H ^+^ so that the transport reaction is driven by the negative bacterial membrane potential and by the transmembrane pH gradient ^67^. The exporter responsible for malate export from the plant across the SM has not yet been identified. There are excellent candidates for dicarboxylate export into the peribacteroid space. Malate is transported by S-type anion channels ^69^ in the SLAC family and by aluminum-activated malate transporters (ALMTs) ^70^. ALMTs were first identified in wheat and were shown to export malate in response to exposure to aluminum. In *Lotus japonicus*, LjALMT4 transports malate and is expressed in nodules but is localized to the vasculature in nodules ^71^. Perhaps another member of the ALMT family localizes to the PBM in infected cells as indicated in Figure 5.

The form of N transported from the bacteroid to the plant is thought to be ammonium (either NH _4_^+^ or NH _3_). Although both plants and bacteria encode ammonium transporters in the AMT/MEP/Rh family ^62^, the current view is that transporters in the AMT/MEP/Rh family are not involved in either NH _3_ release from bacteroids or uptake into infected plant cells. The bacterial AMT *AmtB* is not expressed in bacteroids and therefore N is thought to be exported from bacteroids by diffusion of NH _3_ through the bacteroid membrane ^64^. The aquaporin homolog NOD26 has been suggested to serve as the ammonium uptake transporter from the peribacteroid space into rhizobia-infected cells. NOD26 is highly expressed, localized to the PBM, and known to transport NH _3_^72^. Transporters in the AMT1 family were not identified in proteomic studies of nodules ^73, 74^. GmSAT1 from soybean was originally considered a candidate for the plant PBM ammonium uptake transporter; however, GmSAT1 was subsequently found to be a transcription factor that affects expression of the yeast *MEP3* ammonium transporter when expressed in yeast ^75^.

Since the bacteroid is completely enclosed by the plant SM, it is necessary for the plant to provide all nutrients required by the bacteroid. Recent results demonstrate that uptake into nodule cells is an essential step in providing nutrients to the bacteroid. Molybdenum is required for the nitrogenase enzyme, and in Medicago MtMOT1.3 ^76^ and MtMOT1.2 ^77^ localize to the plasma membrane in nodule cells and function in molybdate uptake. The molybdate exporter, required to deliver molybdate into the peribacteroid space, has not been identified. Synthesis of nitrogenase in the bacteroid requires a significant amount of sulfur (S) for both S-containing amino acids and Fe-S complexes. Bacteroids have been shown to take up 20-fold times more S than the nodule host cells ^78^. The plant SO _4_^2−^ transporter SST1 is required for symbiotic N fixation ^79^, and *sst1* mutant nodules show less S accumulated in host cells, in the symbiosome space, and in bacteroids ^78^. SST1 protein was identified by mass spectroscopy in PBM fractions ^73, 74^; however, its localization has not been confirmed by a second method. SST1 belongs to the SULTR family of plant electrogenic H ^+^-coupled SO _4_^2−^ uptake transporters, so the function of SST1 is most likely S uptake into plant cells in the nodule rather than SO _4_^2−^ export to the bacteroids. If SST1 functions in SO _4_^2−^ uptake, then its localization is more likely to be on the plasma membrane rather than the PBM. Other plant nutrient uptake transporters localized to the plasma membrane, such as the phosphate transporter GmPT7 from soybean ^80^, have been shown to be necessary for optimal N fixation and they also function in uptake into nodule cells rather than nutrient export to the bacteroid ( Figure 5).

The biochemical reactions of N fixation that occur in the bacteroid require transition metals. For example, nitrogenase is the most abundant protein in bacteroids and contains 32 iron atoms and one molybdenum. Cobalt, copper, manganese, nickel, and zinc are also required and must be supplied to the bacteroid. In *Medicago truncatula*, iron transport into rhizobia-infected cells is thought to occur via MtNramp1, which is localized to the plasma membrane in nodule cells ^81^. The *nramp1-1* mutant has lower nitrogenase activity, indicating that less iron is delivered to the bacteroids in the mutant. MtSEN1 may serve as the iron exporter on the PBM necessary for iron transport into the peribacteroid space ^82^. MtSEN1 is in the VIT1/CCC1 family of vacuolar iron exporters that function as H ^+^-coupled antiporters ^83^. Subcellular localization of MtSEN1 will be necessary to support this hypothesis. There is a recent review of transporters involved in export of transition metals from the plant and uptake into the bacteroid ^84^.

## Conclusions

In this review, we discussed five nutritional symbioses and some common features were found; for example, all of the interactions involve the transfer of fixed C and fixed N. When the form of C exported from a photosynthetic partner is sucrose or its hydrolysis products, glucose and fructose, the type of transporter is likely to be a SWEET. The legume–rhizobia interaction is an exception in that C is transferred in the form of the C _4_-dicarboxylate malate. Although the transporter responsible for malate efflux has not been identified, plant anion channels are known to transport malate. The most common form of transported N in these interactions is ammonium. Plants encode both electrogenic (AMT1) and electroneutral (AMT2) ammonium transporters. One common hypothesis is that AMT1s serve as uptake transporters and AMT2s serve as exporters ^62^, but the situation seems to be more complicated and interesting; for example, *AMT2-4* from *Azolla* has been suggested to be involved in ammonium uptake into the fern. This is an exciting time to study symbiotic interactions because of advances in genome sequencing, transcriptomics, and gene editing. Identification and analysis of the transporters involved will allow us to better understand how symbiotic organisms share nutrients.
